# A functional ultrasound brain GPS for automatic vascular-based neuronavigation

**DOI:** 10.1038/s41598-021-94764-7

**Published:** 2021-07-26

**Authors:** M. Nouhoum, J. Ferrier, B.-F. Osmanski, N. Ialy-Radio, S. Pezet, M. Tanter, T. Deffieux

**Affiliations:** 1 Physics for Medicine, INSERM U1273, ESPCI Paris, CNRS UMR 8063, PSL Research University, 17 rue Moreau, Paris, France; 2 Iconeus, 6 rue Jean Calvin, Paris, France

**Keywords:** Biomedical engineering, Neuroscience, Ultrasound, Preclinical research, Imaging

## Abstract

Recent advances in ultrasound imaging triggered by transmission of ultrafast plane waves have rendered functional ultrasound (fUS) imaging a valuable neuroimaging modality capable of mapping cerebral vascular networks, but also for the indirect capture of neuronal activity with high sensitivity thanks to the neurovascular coupling. However, the expansion of fUS imaging is still limited by the difficulty to identify cerebral structures during experiments based solely on the Doppler images and the shape of the vessels. In order to tackle this challenge, this study introduces the vascular brain positioning system (BPS), a GPS of the brain. The BPS is a whole-brain neuronavigation system based on the on-the-fly automatic alignment of ultrafast ultrasensitive transcranial Power Doppler volumic images to common templates such as the Allen Mouse Brain Common Coordinates Framework. This method relies on the online registration of the complex cerebral vascular fingerprint of the studied animal to a pre-aligned reference vascular atlas, thus allowing rapid matching and identification of brain structures. We quantified the accuracy of the automatic registration using super-resolution vascular images obtained at the microscopic scale using Ultrasound Localization Microscopy and found a positioning error of 44 µm and 96 µm for intra-animal and inter-animal vascular registration, respectively. The proposed BPS approach outperforms the manual vascular landmark recognition performed by expert neuroscientists (inter-annotator errors of 215 µm and 259 µm). Using the online BPS approach coupled with the Allen Atlas, we demonstrated the capability of the system to position itself automatically over chosen anatomical structures and to obtain corresponding functional activation maps even in complex oblique planes. Finally, we show that the system can be used to acquire and estimate functional connectivity matrices automatically. The proposed functional ultrasound on-the-fly neuronavigation approach allows automatic brain navigation and could become a key asset to ensure standardized experiments and protocols for non-expert and expert researchers.

## Introduction

In recent years, the advent of ultrafast ultrasound imaging at thousands of frames per second has allowed ultrasound to gain two orders of magnitude of sensitivity to small blood flows^1,2^. This new capability to image small cerebral vessels allowed ultrasound to enter the field of neuroimaging, using cerebral blood volume to measure indirectly brain activation through the neurovascular coupling. Functional Ultrasound (fUS) can provide functional information, such as activation maps or functional connectivity^3^ in the brain, with high spatial and temporal resolution. In preclinical imaging, a large number of studies have been published from small to large animal models demonstrating its potential in neuroscience and clinical research^4^. These studies have been carried out mainly with fUS neuroimaging scanners driving linear ultrasonic probes. Such approach allows high-sensitivity acquisition and to image directly through the skull without complex animal preparation in mice, the most used animal in neuroscience research. Several approaches have been proposed to tackle the challenging whole-brain 3D imaging in fUS, either based on moving linear arrays^5^, matrix arrays^6,7^ or RCAs (raw column arrays)^8^. However, both matrix arrays and RCAs have limited sensitivity, which is not sufficient to perform transcranial imaging even in mice, and therefore require invasive surgery. Thus, high-sensitivity linear probes remain to date the best-suited technology for non-invasive fUS imaging. However, positioning the linear array over the correct slice to image remains challenging. Unlike MRI, ultrasound imaging provides poor anatomical images, especially through the skull, but provides excellent mapping of the vascular anatomy^9^. Although experts can learn after a long training curve to navigate through vascular images and position the array correctly with respect to functional brain areas, there remains a crucial need for a robust and automated approach to lower this know-how barrier, as well as to improve standardization and reproducibility between different operators and different animals or experiments.

In this paper, we investigate how the Doppler contrast, by producing a vascular fingerprint, can be used ‘on the fly’ to provide accurate neuronavigation capability within functional ultrasound experiments.

## Brain positioning principle

**Figure 1:**
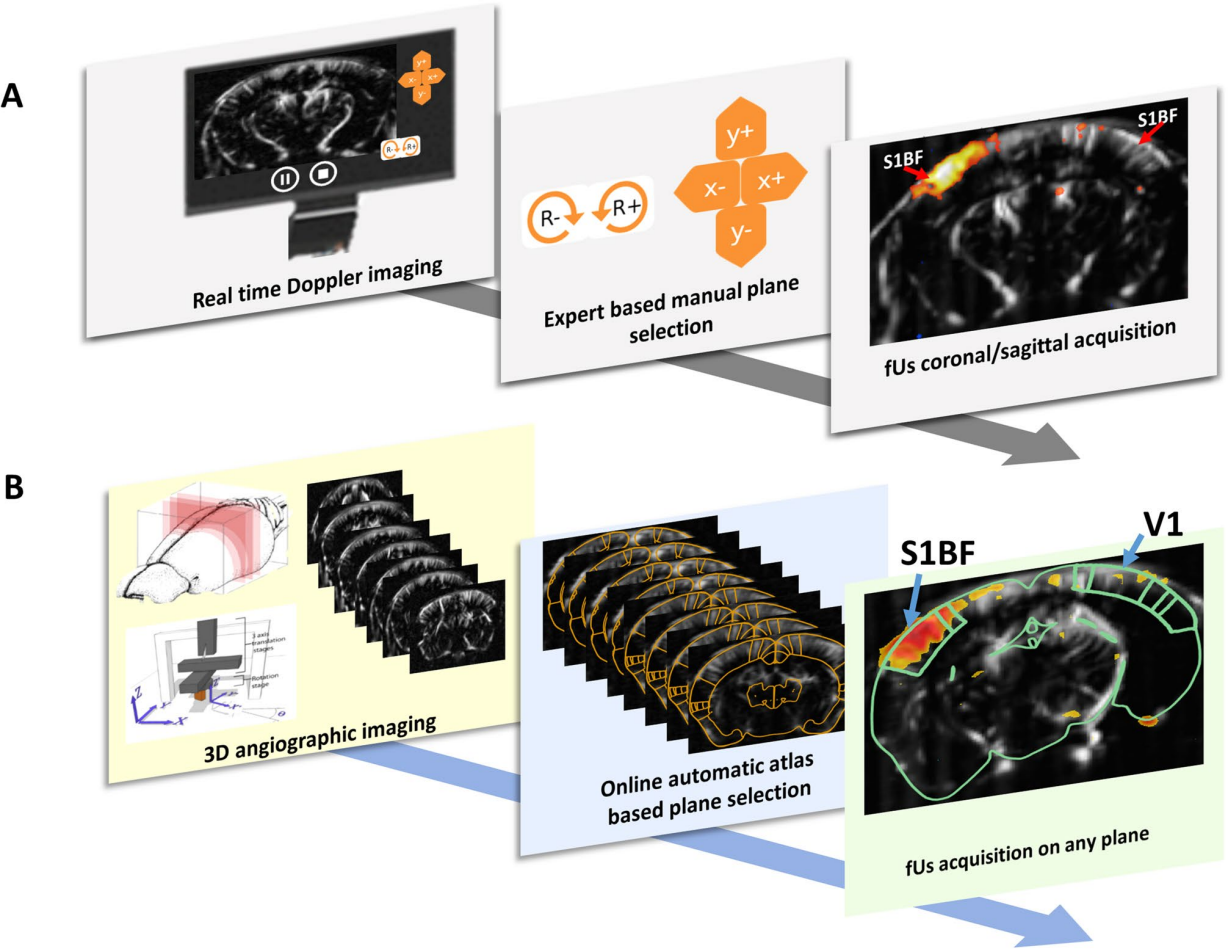
Functional ultrasound imaging workflow. Schematics illustrating the major steps required for the positioning of the ultrasound probe in a dedicated structure. (**A**) Conventional expert-based manual probe positioning is performed by visual recognition of the vascular structures from real-time imaging and motorized setup actioned step by step by the expert (**A**, middle panel). Typically, only canonical planes are recognized and selected (a coronal slice for instance, due to symmetry of brain structures). (**B**) The BPS system performs first a 3D angiogenic scan, followed by an automatic and online atlas-based positioning on any slice from 3D multi-slice Power Doppler angiographic acquisition. S1BF: primary sensory cortex, barrel field part. V1: primary visual cortex.

**Figure 2:**
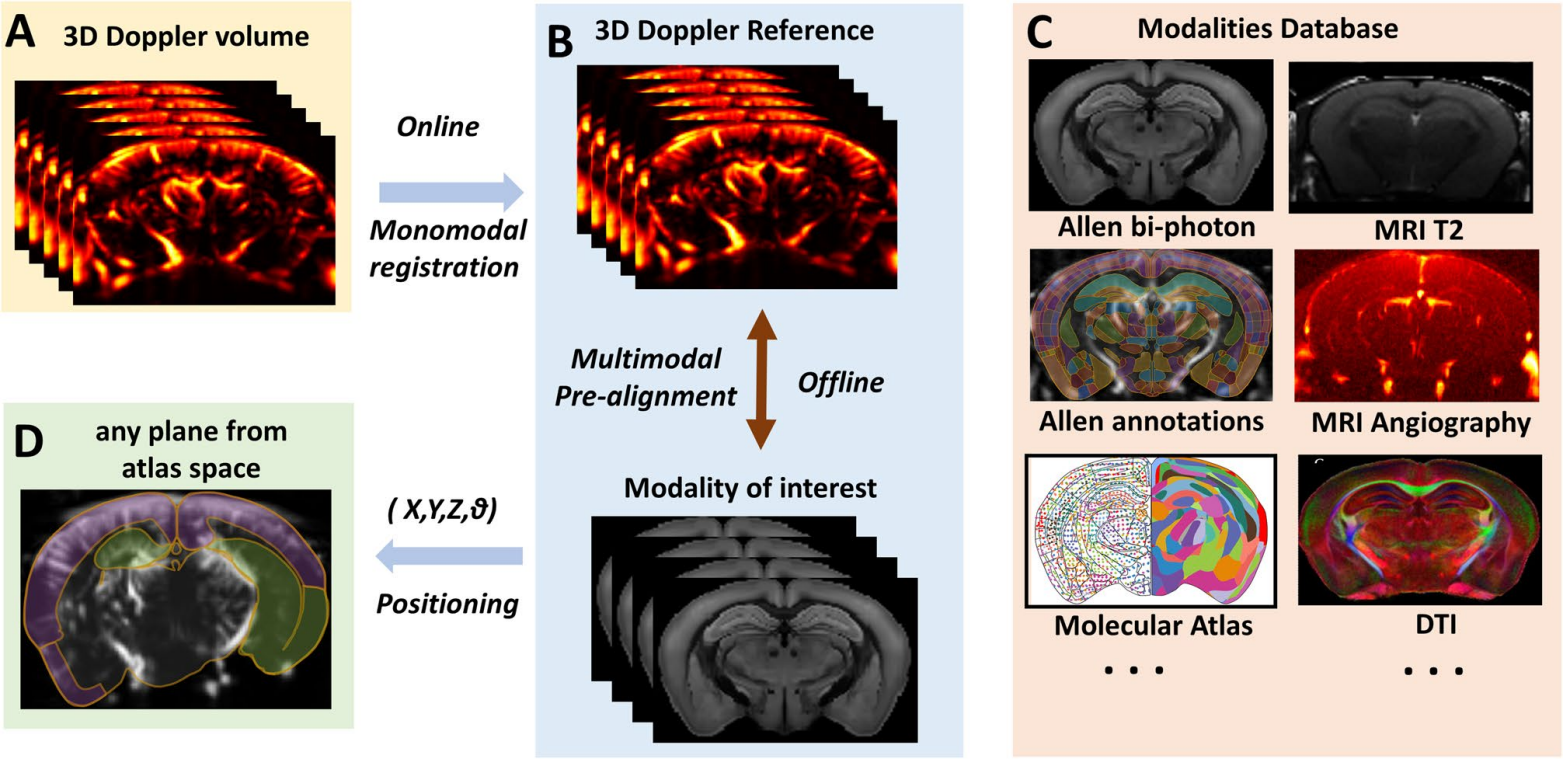
Schematic description of Brain Positioning System principle. A 3D Doppler Volume acquired at the beginning of the imaging session is automatically registered online to the 3D Doppler Reference (**A**) which was pre-aligned offline with a modality of interest, here the Allen Mouse Brain Common Coordinates Framework Atlas (**B**), but could be any other modality providing structural or functional information (**D**). As a result, the anatomic information provided by the modality can be propagated to the fUS experimental framework and used for automatic delineation of the vascular landmarks and atlas-based positioning (**D**).

A classical functional ultrasound imaging experiment starts by positioning the ultrasonic probe over the targeted functional regions^10,11^. For this, users take advantage of the high sensitivity of ultrafast Doppler imaging to reveal and identify vascular landmarks such as vessel shape or branching within the mouse brain. Using a motorized system, they move the probe over different brain areas and image several canonical planes until they recognize those vascular clues, usually restricting themselves to coronal slices, which are much easier to apprehend (Fig. 1A). They can then perform functional ultrasound acquisition with high frame rate either on a single slice or on several slices^5,12^. fUS imaging remains thus inherently limited by the expertise required to neuronavigate oneself within the brain prior to acquisition. The brain positioning system (BPS) proposed here provides a fully-automatic approach to solve this problem during the experiment, and provides a familiar anatomical context and automatic probe positioning in any desired plane and orientation for the whole mouse brain (Fig. 1B). Our approach relies on the coupling of an online automatic registration (Fig. 2A) of a 3D Doppler volume—acquired at the very beginning of the imaging session—to a 3D Doppler reference, which was pre-aligned once with any modality of interest (Fig. 2B, C). This modality can for instance provide anatomical or functional information, and we illustrate mainly in this work the use of the Allen Mouse Brain Atlas^13^ to search, display and finally reach specific structures to image (Fig. 2D), thanks to the coupling with a motorized system.

Many studies have explored the possibilities of using vascular information to register volumes either within or between modalities^14–16^. In clinical neuroscience, an active and strong area of research has been to explore brain shift correction during intraoperative surgery in order to correct brain MR images from live Doppler data^14^. By leveraging the brain vascular footprints and integrating the approach directly in the functional ultrasound experimental workflow, we can improve the quality and standardization of the experimental data. In that regard, our ‘on the fly’ BPS approach brings functional ultrasound imaging closer to fMRI, where MR images can be used to position acquisition slices, and MR contrast templates can be used by software suites such as SPM or FSL to wrap and normalize the datasets to a common space for group-level analysis^17,18^.

We first illustrate the specificities and invariants of the registration of those vascular footprints obtained from 3D Doppler volumes by evaluating intra-animal and inter-animal pairs of images and resulting cross-correlation profiles. We then compare the automatic BPS registration results with landmark-based manual registration positioned by trained experts with more than 40 cumulative years in neuroscience and neuroanatomy. Finally, in order to evaluate the accuracy of the Doppler registration, we use BPS-aligned super-resolution angiographic images with tens of micrometers spatial resolution to evaluate positioning errors with tens of micrometers accuracy.

The BPS is then evaluated for functional activation mapping in complex, oblique planes, and for functional connectivity assessment, with the added benefit of automatic extraction of regions of interest.

## Materials and methods

Methods were carried out in accordance with relevant guidelines and regulations and in compliance with ARRIVE guidelines.

### Animals

All animals received humane care in compliance with the European Union Directive of 2010 (2010/63/EU), and the study was approved by the institutional and regional committees for animal care: CEEA (Comité d’Ethique pour l’Expérimentation Animale) numéro 59—‘Paris Centre et Sud’ Protocole # 2017-23). Twenty-four adult mice (male C57BL/6 Rj, 8–14 weeks old, average weight 25.6 ± 1.5 g from Janvier Labs, France) were used for this study. On arrival, they were housed 4 per cage with a 12 h light/dark cycle, constant temperature at 22 °C and food and water ad libitum. Before the beginning of the experiments, animals were given a 1-week minimum acclimatization period to housing conditions. All animals used in this study were untreated. They were assigned randomly in the different experiments. We did not exclude any animal from our analysis.

### Anesthesia and animal preparation

Animals were anesthetized by intraperitoneal injections of a bolus of Ketamine (60 mg/kg) and Medetomidine (1 mg/kg). Once anesthetized, animals were placed in a stereotaxic frame (David Kopf, Tujunga, USA). After shaving the hair on the head using a depilatory cream (Clarins, France), echographic gel was applied and the ultrasound probe was then placed in contact with the gel. Imaging was therefore performed through the skin and skull. Anesthesia was maintained by subcutaneous infusion of Ketamine (20 mg/kg/h), Medetomidine (0.3 mg/kg/h) using a push syringe (KD Scientifics).

### Ultrafast ultrasound power Doppler imaging sequence

Transcranial ultrafast acquisitions were performed using the Iconeus One scanner (Iconeus, Paris, France) driving a 15–20 MHz probe (128 elements, 0.11 mm pitch) mounted on a motorized setup. Each image at a unique position of the probe was obtained from 200 compounded frames acquired at 500 Hz frame rate. Each of these raw ultrasonic frames was built using 11 tilted plane waves (− 10°, − 8°, − 6°, − 4°, − 2°, 0°, 2°, 4°, 6°, 8°, 10°) acquired at 5500 Hz pulse repetition frequency. Power Doppler images were obtained after blocks of 200 compounded frames were processed using a spatiotemporal Singular Values Decomposition (SVD) clutter filter^19^ to discriminate blood flow from tissue motion signal. The imaging device enables 100 μm × 100 μm in plane resolution with a slice thickness of approximately 400 μm. Successive 2D slices were acquired with 0.2 mm spatial steps to reconstruct 3D vasculature with (100 × 100 × 400) μm^3^ resolution. Functional ultrasound imaging sessions were performed by real-time continuous acquisitions of successive blocks of 400 ms (200 compounded frames at 500 Hz), resulting in a 2.5 Hz Doppler frame rate.

### Ultrafast ultrasound localization imaging

Initial suspension of Sonovue microbubbles solution (Bracco Spa, Milan, Italy) was obtained by dissolving 2.5 mg powder in 5 mL NaCl solution. 70 μL of the initial solution was injected through the catheter placed in the tail vein of the mouse. Iconeus One scanner was used to acquire 600 blocks of 400 compounded frames acquired with 9 angles (− 8°, − 6°, − 4°, − 2°, 0°, 2°, 4°, 6°, 8°) at a 1000 Hz frame rate. Microbubble signals were separated from the surrounding tissue signal using an SVD filter. Microbubbles were tracked using an algorithm based on the Hungarian method^20^. Each track was smoothed with sliding average, interpolated and projected on a 2D grid to reconstruct the density and velocity maps of the microbubbles^21,22^.

### Reference Doppler and brain template offline alignments

A transcranial 3D Doppler volume was first acquired using the ultrafast Doppler tomographic approach^9^ using the same parameters as described in the Power Doppler section with 19 rotations. Briefly, high-sensitivity Doppler images of the mouse brain were acquired at different positions and angles and used to reconstruct an isotropic high-resolution 3D volume of the vascular network. In this work, this Doppler volume was then wrapped to the Allen Atlas two-photon template using the elastix module of the slicer3D software^23–25^ using the Dorr vascular atlas^26^ as an intermediate. This Doppler volume was first manually registered to the Dorr CT angiography and the Allen two-photon template to the Dorr T2 MRI. Each registration operation was performed semi-automatically with an initial manual registration by expert neuroanatomists using manual landmarks, then automatic registration using a non-rigid bspline-based algorithm with slicer3D. Note that vascular references could also be aligned using MR angiography scans as a bridge between Doppler data and Atlas templates.

### On-the-fly vascular registration and neuronavigation

During the experiments, a 3D Doppler volume was acquired and then automatically registered using a dedicated prototype software.

First, the 3D Doppler volume was used to automatically perform an affine monomodal registration with the 3D Doppler reference. The chosen registration algorithm is based on Mattes mutual information metric maximization^27,28^ to measure how similar the images are, and an evolutionary optimizer^29^ to find at each iteration a set of parameters that produce the best registration result (Mathworks, Natick, Massachusetts, USA). To ensure the reproducibility of registration, all the spatial samples were used to compute the probability density over 50 bins, enabling mutual information estimation between the two data sets. The software then automatically applied the geometric transformations from the Allen Atlas space to the 3D Doppler volume through the 3D Doppler reference to overlay the Allen structures directly on the 3D Doppler volume and to successively acquired scans. For neuronavigation and automatic positioning of the probe, the software is used to define a new virtual imaging plane from the position of two markers that can be set by the user on top of the overlaid brain structures. The motor positions of the robotic platform are then computed automatically from the plane coordinates with an inverse kinematic solver (*inverseKinematics*, Mathworks) and are used to move the probe over the desired imaging plane.

### Registration accuracy estimation from Power Doppler images

3D linear scans were acquired with *n* = 5 C57BL/6 mice for Power Doppler based registration estimation. Each scan has a 6 mm antero-posterior range covering the entire Bregma–Lambda region with (100 × 100 × 400) μm^3^ resolution.

#### Normalized cross-correlation

To illustrate the similarity between the registered volumes, we computed the 3D normalized cross-correlation of the Doppler volumes. We then measured the peak value, peak location and peak width as simple metrics of volumes similarity, shift and spatial precision. Please note that the correlation is not used for the registration but only for illustration.

### Registration accuracy estimation from the comparison of the BPS with manually positioned vascular landmarks

#### Definition of vascular landmarks

A common set of vascular landmarks were chosen by two experts in the field and can be seen in supplementary Fig. 1.

The first vascular landmark VL1 (β − 1.25 mm) is located at the junction between right and left aspects of the medial choroid plexus (yellow arrow). The plane can be recognized by the circular shape of the choroid plexus (arrow 1) and artery in the ventral aspect of the brain (arrow 2).

The second vascular landmark VL2 (β − 1.85 mm) is the change of direction in the AchA (yellow arrow), from angle to vertical. The plane is recognizable by the presence of the AchA^26^ (anterior choroidal artery, arrow 1) and the internal carotid (arrow 2).

The third vascular landmark VL3 (β − 2.45 mm) is located on the right ending of the medial pointy part of the choroid plexus (yellow arrow). The plane is recognizable by the round and descending aspect of the thalamic artery (arrow 1) and presence of the AchA (arrow 2).

The fourth vascular landmark VL4 (β − 2.65 mm) is the medial and most ventral joining point of PCA (posterior cerebral artery) medially (yellow arrow). The plane is recognizable by the PCA. Note that (a) the planes containing the markers 3 and 4 are very close and sometimes in the same plane of imaging, and (b) the marker 4 is not the joining point, which is a few millimeters more dorsally (arrow 2).

#### Registration and resampling

We first considered a group of 5 acquisitions (3D Power Doppler with 100 × 100 × 400 μm^3^ resolution; 3 days between the first and the last acquisition) from the same animal to evaluate longitudinal intra-animal registration. Successively, each of the acquisitions was used alternatively as a reference volume to register the other acquisitions, resulting in 20 registration operations. Moved volumes were obtained after registration and resampling within the reference space.

#### Manual annotation of registered volumes using the chosen vascular landmarks

For each pair of acquisitions (reference and moved volumes), four vascular landmarks are manually labeled within each volume by two experts. For each landmark a pair of coordinates is then recorded in the reference space.

#### Discrepancies between BPS estimation and neuroanatomists’ manual annotations

Since the moved volume is resampled within the reference space, for each landmark any difference between the two values is an estimate of the shift between the BPS automatic registration prediction and the experts’ annotation. These discrepancies are computed as 3D distance shifts and averaged over all the pairs of registration operations.

#### Discrepancies between neuroanatomists’ manual annotations

Since the two experts’ manual annotations were performed under the same conditions, raw differences between recorded coordinates for both experts allows estimation of any discrepancies between the manual annotations. These discrepancies are computed as 3D distance shifts and averaged over all the registration operations.

For inter-animal assessment, the same method was performed with a second group including 5 acquisitions from 5 different mice. A total number of *n* = 5 mice were used for this estimation.

### Registration accuracy estimation with ULM super-resolution images

Finer accuracy measurements of the vascular registration were performed using super-resolution images of the vascular structures using ULM^21^. The BPS was used to position the probe and perform an initial Doppler and ULM acquisition in specific slices. In a second, independent experiment, the BPS was used again to reposition the ultrasonic probe on the very same imaging plane, and both new Power Doppler and ULM images were acquired. The reference volume for the BPS could be either from the same animal (direct registration) or from another previous animal (indirect registration). The direct registration was intended to evaluate the vascular-to-vascular registration accuracy with the same animal as used in a longitudinal study, while the indirect registration evaluates the BPS accuracy with a pre-aligned template acquired from a different animal. The super-resolved images from the first and second experiments were then compared to measure the misalignment (as 2D translations) due to the BPS in both coronal and sagittal planes. For each pair of super resolution images, a displacement map is computed in both lateral and axial directions (supplementary Fig. 2) using non parametric non-rigid^30,31^ registration. The displacement is averaged over the entire vasculature to provide single misalignment value for each direction from this pair of acquisitions. This operation was repeated for 15 pairs of coronal acquisitions (X,Z directions) and 7 pairs of sagittal acquisitions (Y,Z directions) performed in *n* = 3 C57BL/6 mice for the direct registration group. The indirect registration group included 20 pairs of coronal acquisitions and 10 pairs of sagittal acquisitions performed in *n* = 6 C57BL/6 mice. A total of n = 9 mice were used for ULM experiments.

#### Task-evoked functional imaging

A first marker was set in the center of the left primary somatosensory, barrel field area and a second marker in the center of the right primary visual area (Fig. 6, red box). This allows automatic probe positioning on an oblique imaging plane which encompasses both functional areas targeted. Then we successively performed whisker stimulation and visual stimulation. A few posterior and caudal right whiskers were stimulated with the following pattern: 30 s baseline followed by three consecutive trials of 30 s ON and 30 s OFF for a total acquisition duration of 210 s. A red LED was placed at 3 cm in front of the left eye to perform visual stimulation with the same pattern as for whiskers.

Activation maps were obtained by computing *Z*-scores using a generalized linear model analysis (GLM) between each voxel temporal signal and the stimulus pattern. Thus multiple hypotheses are tested to compare the entire imaging area and a conservative Bonferroni correction is applied by dividing the overall conventional *p*-value (0.05) by the number of independent hypotheses (number of voxels) to compensate for the increase of type I error. Therefore the individual voxel activation is tested with *p*-value < 0.00001 level of significance. *Z*-score maps are overlaid on the baseline Power Doppler image. Anatomical regions from the Allen Mouse Brain Common Coordinate Framework are automatically positioned and displayed for reference by the BPS. A total of *n* = 5 mice were used for task-evoked functional imaging.

#### Functional connectivity analysis

Under stable conditions, three acquisitions of 600 s at different slices were performed for resting-state connectivity analysis. A low-pass filter with 0.1 Hz cutoff frequency was used to select spontaneous low-frequency CBV (cerebral blood volume) fluctuations. We used the BPS to automatically register the acquisitions to the Allen Brain Atlas. This enables the identification and automatic extraction of about 70 regions of interest based on the Allen ontology grouped in larger regions (isocortex, thalamus and hypothalamus). The connectivity matrix is obtained by computing the normalized Pearson correlation between spatial averaged and temporal filtered signals extracted from each selected ROI. The correlation coefficients are color-coded for representation. For seed-based analysis, several seeds identified from the automatic registration on Allen Brain Atlas were selected. Seed-based correlation maps were formed by computing the normalized Pearson correlation coefficient between the average signal of the seeds and each individual voxel of the data. The correlation coefficients were color-coded and overlaid on the baseline Power Doppler image. Anatomical regions from the Allen Mouse Brain Common Coordinate Framework are displayed including the seed region (with different color). A total number of *n* = 5 mice were used for functional connectivity imaging.

## Results

### Automatic registration is as accurate as neuroanatomist expert annotation

**Figure 3:**
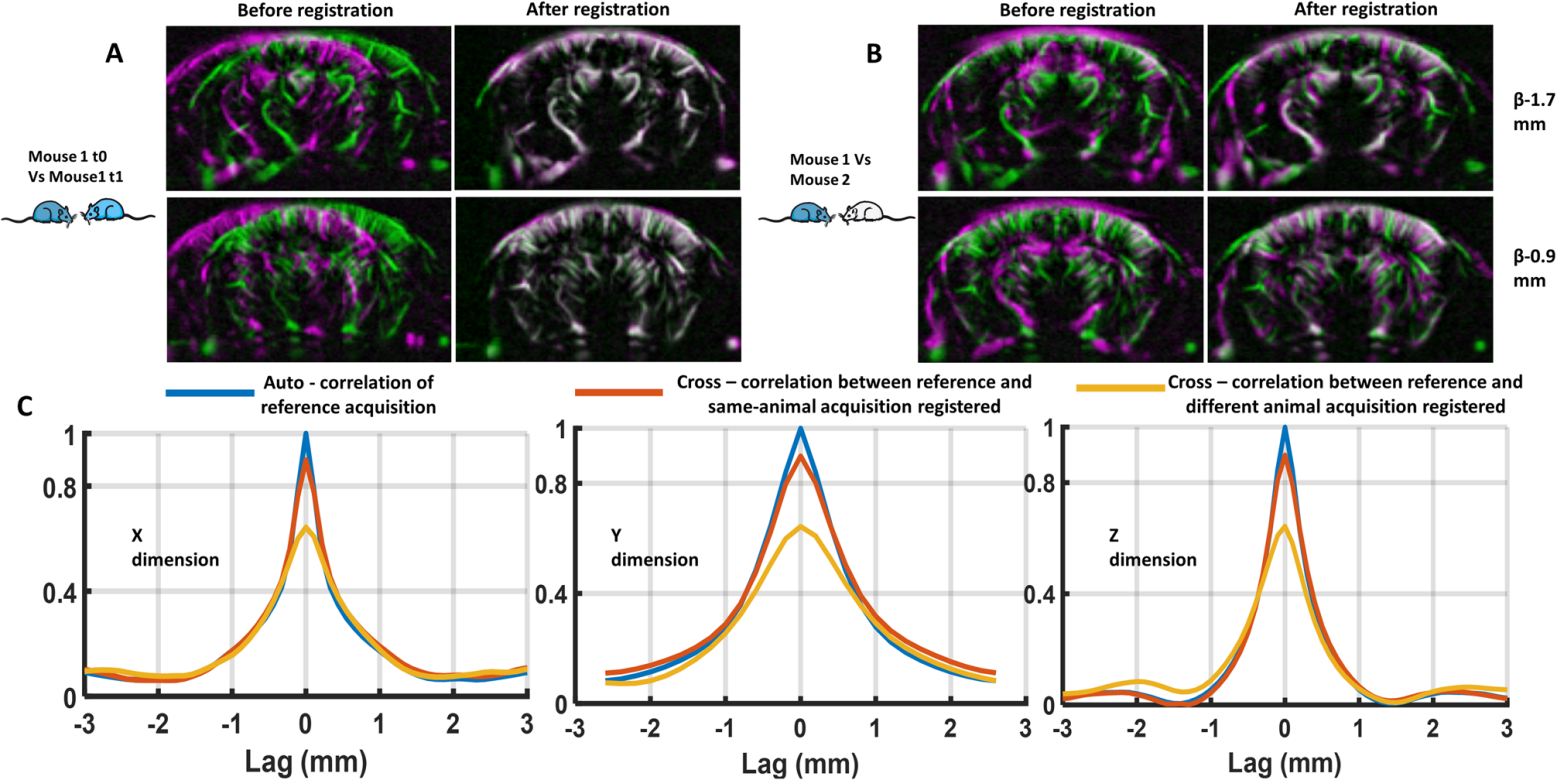
General assessment of Power Doppler angiographic image registration. (**A**) Typical example of vascular images obtained in a pair of acquisitions from the same animal acquired at different time points, before registration (left) and after registration (right). Color code: Green T0, magenta T1. (**B**) Same representation as (**A**), for a pair of acquisitions acquired in different animals. In A and B, images on the top row were obtained at coordinates Bregma − 1.7 mm, and at Bregma − 0.9 mm on the bottom row. (**C**) Cross-correlation plot between reference data and registered data in the three space directions. Reference data auto-correlation is also shown.

The accuracy of the vascular registration is a critical point in the proposed neuronavigation approach. We first assessed the accuracy of the ultrafast Doppler vascular-to-vascular volume registration in the same mouse at different time points. Inherent spatial shifts between acquisitions is inevitable during the experiment and the mouse installation in the stereotactic frame. The green–magenta representation illustrates this initial misalignment between the two datasets before registration (Fig. 3A). The first acquisition (time point t0) is displayed in green, while the second acquisition from the same animal acquired at time point t1 (24 h later) is considered to be the moving volume (magenta) that we want to register. After registration and resampling of the second volume to the first one, a good match is found by the registration algorithm between the two vascular datasets (Fig. 3A), as illustrated by the dominance of white color. On the same animal, the normalized cross-correlation between the reference and registered volumes shows a strong (value = 0.9) and sharp (width at half-maximum Δ*x* = 0.65 mm, Δ*y* = 1.34 mm, Δ*z* = 0.64 mm) peak located in the center (Fig. 3C). As reference, autocorrelation (same volume) yields a full width at half maximum (Δ*x* = 0.5 mm, Δ*y* = 1.15 mm, Δ*z* = 0.55 mm).

Although the details of the vascular networks are different, registration between different animals is still good because of the general vascular architecture of the larger vessels (Fig. 3B), as illustrated by the white areas located in larger vessels in the green–magenta representation. The normalized cross-correlation between the reference and registered volumes demonstrates a lower correlation (value = 0.64) and a wider (Δ*x* = 1.07 mm, Δ*y* = 1.71 mm, Δ*z* = 0.83 mm) peak, which is expected because the vascular fingerprints are now different and the inherent uncertainty higher.

**Figure 4:**
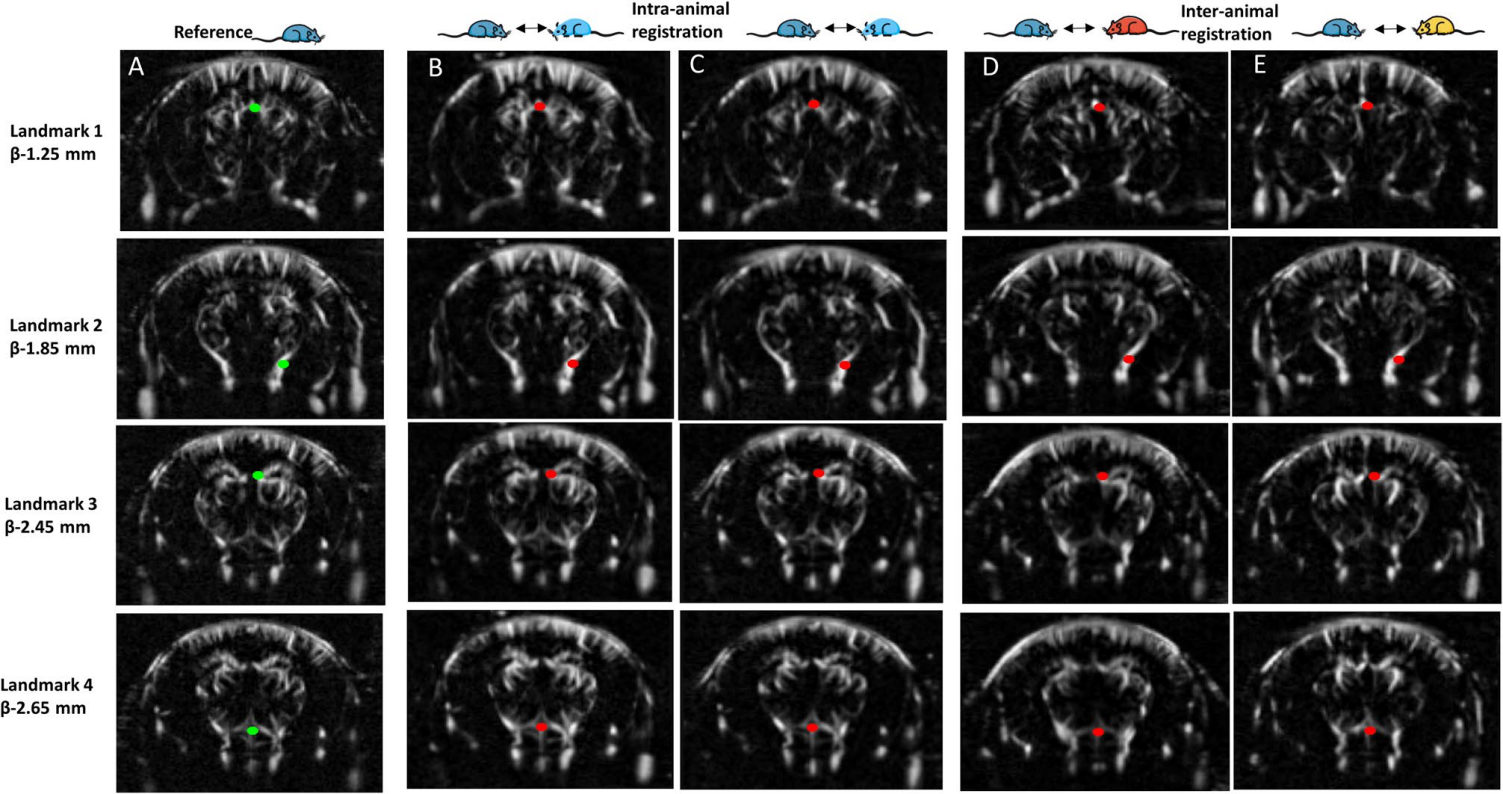
Vascular landmark-based illustration of intra- and inter-animal registration. (**A**) Four vascular landmarks were defined (green points) at four different coronal slices. The detailed description of the landmark is provided in supplementary Fig. 1. (**B**,**C**) Each of the columns illustrates the matching slices from the same-animal acquisitions registered onto the reference. Landmarks predicted by the automatic registration are highlighted in red. (**D**,**E**) Same representation as (**B**,**C**) for acquisitions from two different animals onto the reference dataset. These results highlight the reproducible detection of these landmarks both between sessions in the same animal (intra-animal variability) and between animals (inter-animal variability).

We then evaluated the accuracy of the registration with vascular landmarks. First, the result of registration and resampling automatically performed is illustrated in Fig. 4. Automatic predictions of landmarks placed in the reference (green point Fig. 4A) are illustrated in red in moved volumes for both intra-animal (Fig. 4B,C) and inter-animal registration (Fig. 4D,E). We can note a good reproducibility of registration of these landmarks both between acquisitions in the same animal and between animals.

Then we compared the discrepancies of the landmarks placed by trained experts in different datasets with those automatically predicted by the registration (as described in ‘Materials and methods’). The results are reported in Table 1. First, we compared independently each expert annotation to the BPS registration. Automatic registration had an average shift of 120 ∓ 84 μm (ranging from 78 ∓ 58 to 158 ∓ 94 μm) for the four vascular landmarks compared to the first neuroanatomist’s expert annotation. The second expert annotated the landmarks with an average 130 ∓ 82 μm (ranging from 105 ∓ 70 to 155 ∓ 90 μm) shift compared to the automatic registration. As a comparison, we compared the two experts’ annotations to one another. Their estimations were globally misaligned by 215 ∓ 87 μm (ranging from 184 ∓ 94 to 253 ∓ 101 μm). Thus, we found that for any of the four landmarks and for the overall annotation the discrepancy between experts’ annotations was higher than the one between each individual expert annotations and the BPS prediction. It shows that the accuracy of the automatic registration is well within the expert manual positioning error. Notably, experts needed about 5 min to navigate the slices and annotate the four landmarks within a pair of acquisitions, instead of a minute required by the automatic approach to register the entire brain vasculature.

**Table 1:** Landmark-based comparison between automatic registration predictions and expert neuroanatomists’ manual annotations of these landmarks.

	Intra-animal registrations (*n* = 20)			Inter-animal registrations (*n* = 20)		
	AR vs Expert1	AR vs Expert2	Expert1 vs Expert2	AR vs Expert1	AR vs Expert2	Expert1 vs Expert2
Landmark 1 (µm)	158 ± 94	155 ± 90	184 ± 94	188 ± 83	266 ± 129	297 ± 114
Landmark 2 (µm)	78 ± 58	140 ± 90	191 ± 76	152 ± 78	207 ± 97	214 ± 87
Landmark 3 (µm)	154 ± 82	105 ± 70	253 ± 101	178 ± 81	246 ± 82	269 ± 113
Landmark 4 (µm)	88 ± 69	118 ± 74	233 ± 57	139 ± 66	163 ± 76	255 ± 75
**All (µm)**	**120 ± 84**	**130 ± 82**	**215 ± 87**	**164 ± 78**	**220 ± 104**	**259 ± 102**

20 registration operations were performed for both intra-animal and inter-animal acquisitions between paired acquisitions (inter- or intra-animals). For each pair, the automatic registered data was resampled in the reference dataset space and two expert neuroanatomists were asked to annotate four landmarks within the two datasets. Individual landmark 3D distance shifts between registration prediction and expert annotation were averaged over the 20 estimations, as well as the overall shift. Automatic registration (AR) was compared to individual expert annotation, and the two experts’ annotations were compared to each other. Automatic registration predictions were globally shifted by 120 ∓ 84 μm relative to the first expert annotation and by 130 ∓ 82 μm relative to the second, whereas inter-annotator shift was globally estimated to 215 ∓ 87 μm for inter-animal dataset registration. The same shifts are estimated to respectively be 164 ∓ 78 μm, 220 ∓ 104 μm and 259 ∓ 102 μm for inter-animal dataset registration. The variance was computed as standard deviation.

We then investigated this comparison for inter-animal registration. Predictions from our automatic approach between different animals were shifted by 164 ∓ 78 μm (ranging from 139 ∓ 66 to 188 ∓ 83 μm) compared to the first annotator and by 220 ∓ 104 μm (ranging from 163 ∓ 76 to 266 ∓ 129 μm) compared to the second. The two experts’ estimations were misaligned by 259 ∓ 102 μm (ranging from 214 ∓ 87 to 297 ∓ 114 μm), yielding again a higher inter-expert discrepancy than when compared with the BPS result.

### Ultrasound localization microscopy (ULM) allows evaluation of registration error at micrometric scale

**Figure 5:**
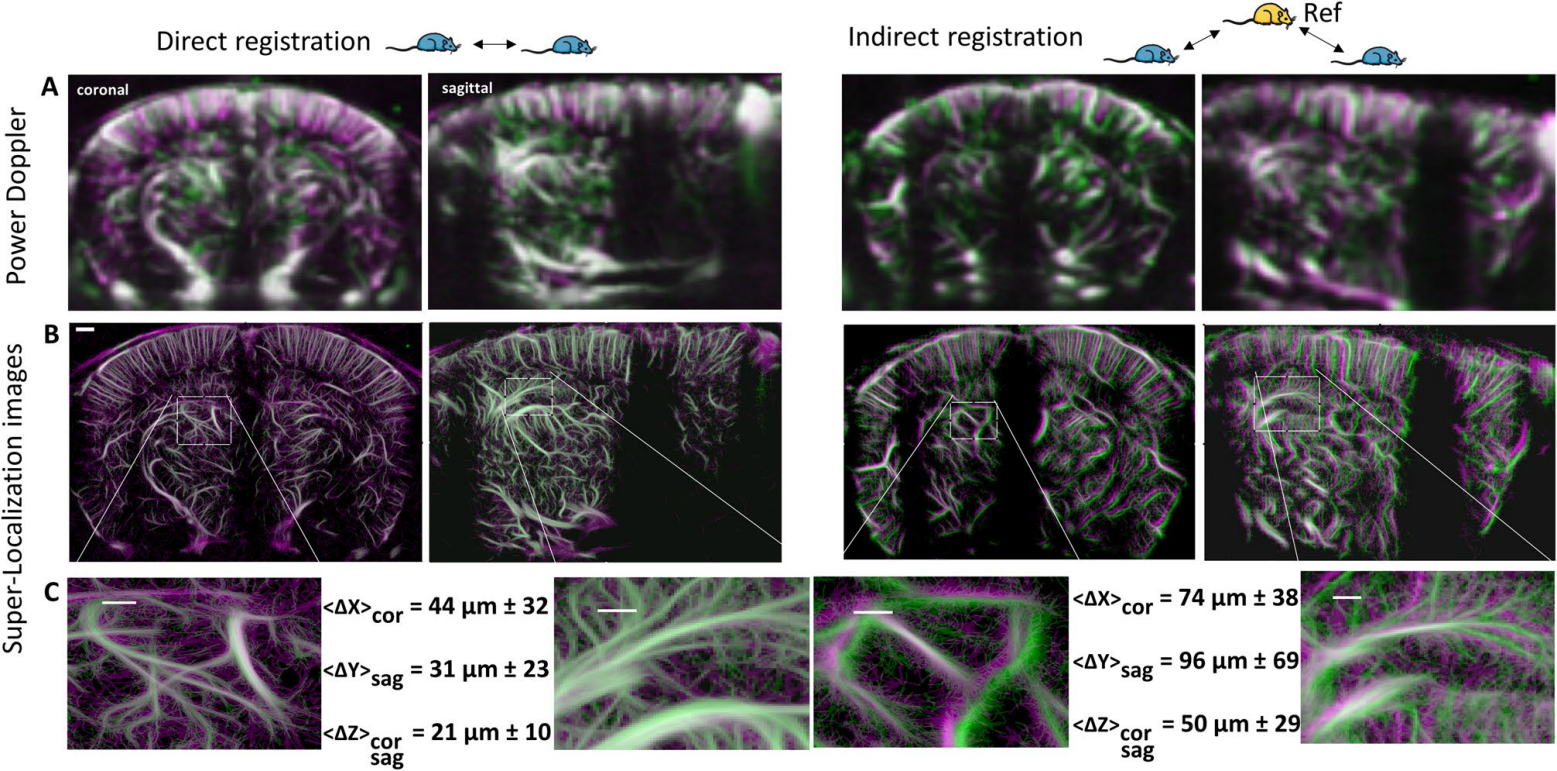
Registration accuracy estimation based on super-localization imaging. Successive and time-delayed registrations are used to position the probe and image the same coronal or sagittal slice. Shifts from reconstructed images from several trials can be estimated as 3D translations enabling the evaluation of the registration process. (**A**) Power Doppler images from a pair of acquisitions are overlaid both in coronal and sagittal directions and for both intra-animal and inter-animal data registrations. Misalignment cannot be correctly estimated with this level of detail (100 μm resolution). (**B**) Corresponding super-localization images, as microbubbles density reconstructed with 5 μm pixels. Scale bar is 500 μm. (**C**) Zooming boxes showing finer local misalignment. Displacement map was computed as 2D translations between a pair of images and averaged over the whole images and over both pairs of acquisitions to evaluate intra-animal and inter-animal data registration accuracy in the 3 space directions as Δ*x* (lateral error from coronal acquisitions), Δ*y* (elevation error from sagittal acquisitions) and Δ*z* (axial error from both coronal and sagittal acquisitions). Scale bar is 200 μm within zoomed boxes.

In order to assess the accuracy of the vascular registrations at an even smaller scale, we further measured the remaining misalignment using super-resolution images after repositioning with the BPS as described in the ‘Materials and methods’ section. One can barely notice misalignment when looking at Power Doppler images of a pair of acquisitions overlaid on each other (Fig. 5A) especially in direct registration trials where we expect lower misalignment. A few shifts in large vessels can be noticed in indirect registration acquisition, which can be due to inherent differences in the vascular architecture of the animals, but the image resolution does not allow a fine estimation of the misalignment. On the other hand, after 3 min of acquisition about 3.10 × 10^5^ bubbles were detected to reconstruct super-resolved coronal and sagittal slices with 5 μm pixel size (Fig. 5B). The images corresponding to the local microbubble density illustrate the increase in spatial resolution on the whole image. These super-resolution images allow us to provide an accurate estimation of the remaining BPS misalignment between a pair of acquisitions. Local vasculature details can be seen within zoomed box regions (Fig. 5C). For each pair of acquisitions the displacement map was averaged over the entire brain region to estimate the registration error for this pair. With a total of 15 pairs of coronal acquisition (X,Z direction) and 7 pairs of sagittal acquisitions (Y,Z direction) we estimated the intra-animal vascular registration error to be (44 ∓ 32, 31 ∓ 23 and 21 ∓ 10) μm respectively in the X (lateral), Y (elevation) and Z (axial) directions. The 12 pairs of coronal acquisitions and the 10 pairs of sagittal acquisition after indirect registration on a reference vasculature from a different animal show an average inter-animal registration error of (74 ∓ 38, 96 ∓ 69 and 50 ∓ 29) μm respectively in the X (lateral), Y (elevation) and Z (axial) directions.

### Task-evoked activation maps

**Figure 6:**
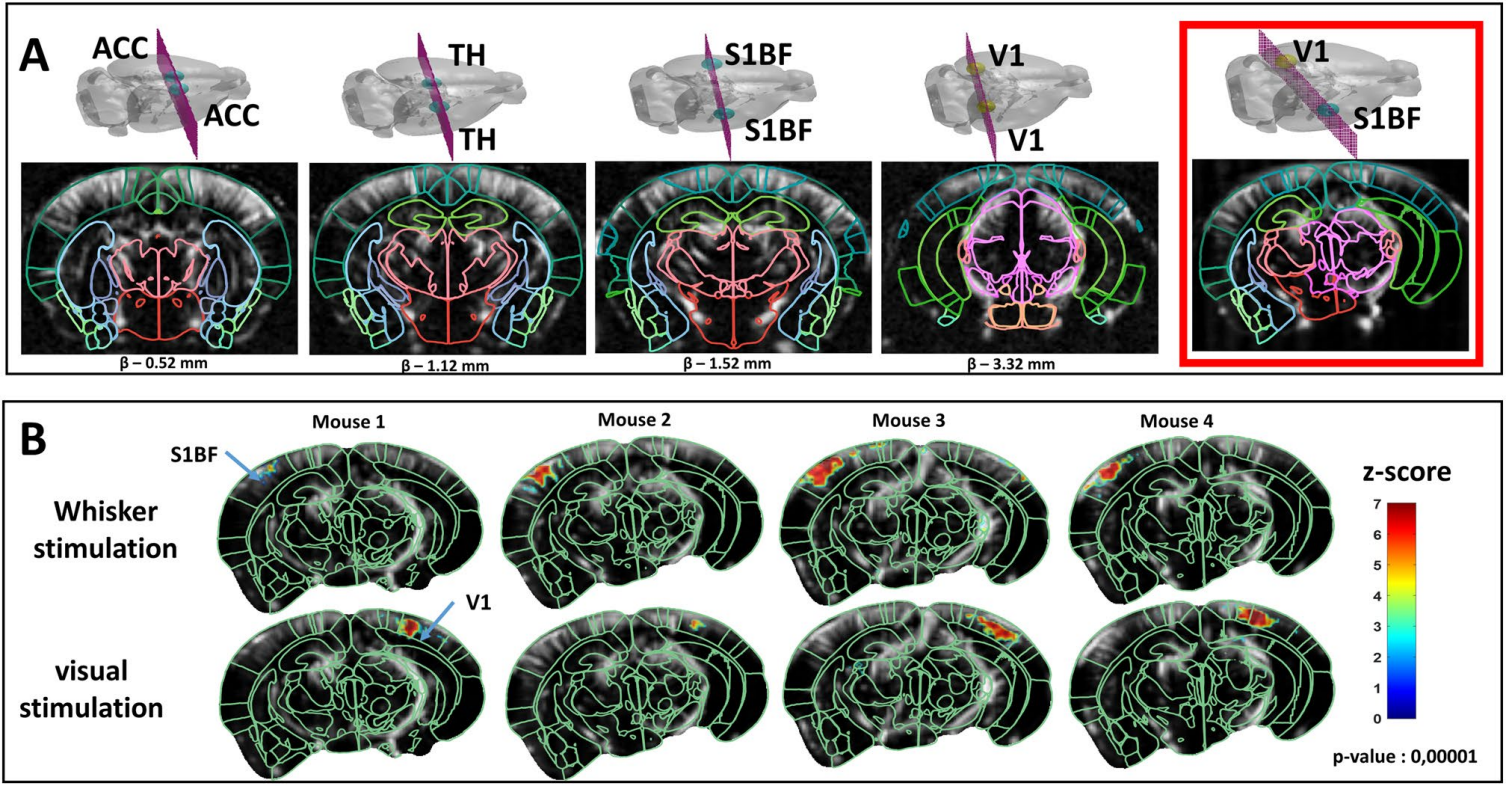
Transcranial functional imaging session using BPS. (**A**) Anatomic labeling guided by Allen CCF (Common Coordinates Framework) atlas on Power Doppler images from online registration. Automatic online positioning is illustrated with the red box. (**B**) Functional imaging after automatic positioning on an oblique plane encompassing both V1 and S1BF. Both whiskers and visual simulations were performed. Activation map obtained with *n* = 4 C57BL/6 mice by computing *Z*-score (color-coded) based on the generalized linear model with Bonferroni correction is superimposed on the baseline Power Doppler image. Automatically aligned anatomic delineations from the Allen CCF are shown in green for reference.

One of the goals of this study was to validate the fully automatic neuronavigation approach proposed here for functional ultrasound imaging application. Here, the goal is to perform functional mapping of the brain activation in a 2D plane containing two chosen regions of interest without the intervention of an expert neuroanatomist. First, the BPS was used to provide whole-brain annotation in the experimental framework (Fig. 6A) enabling whole-brain exploration and real-time anatomic identification. Leveraging this asset, we automatically targeted an oblique plane encompassing both the visual cortex in one hemisphere and the somato-sensory area in the second hemisphere, as labelled in the Allen Atlas (Fig. 6A, red box). After automatically moving the probe to this oblique slice, we then recorded transcranial Power Doppler images during successive right whisker stimulation and left visual stimulation in anesthetized conditions. In *n* = 4 mice for both stimuli, we obtained high and significant (*p*-value = 0.00001 after stringent Bonferroni correction for multiple analysis) *Z*-score values (Fig. 6B). Still leveraging the BPS, the structure contours are automatically overlaid over Power Doppler images and *Z*-score maps, with good agreement between the activated regions and the targeted functional regions. This validates the targeting and automatic probe positioning of the BPS approach to enable functional ultrasound imaging in complex planes for non-experts.

### Functional connectivity analysis

Finally we demonstrated our neuronavigation approach capability for the acquisition and analysis of functional connectivity.

**Figure 7:**
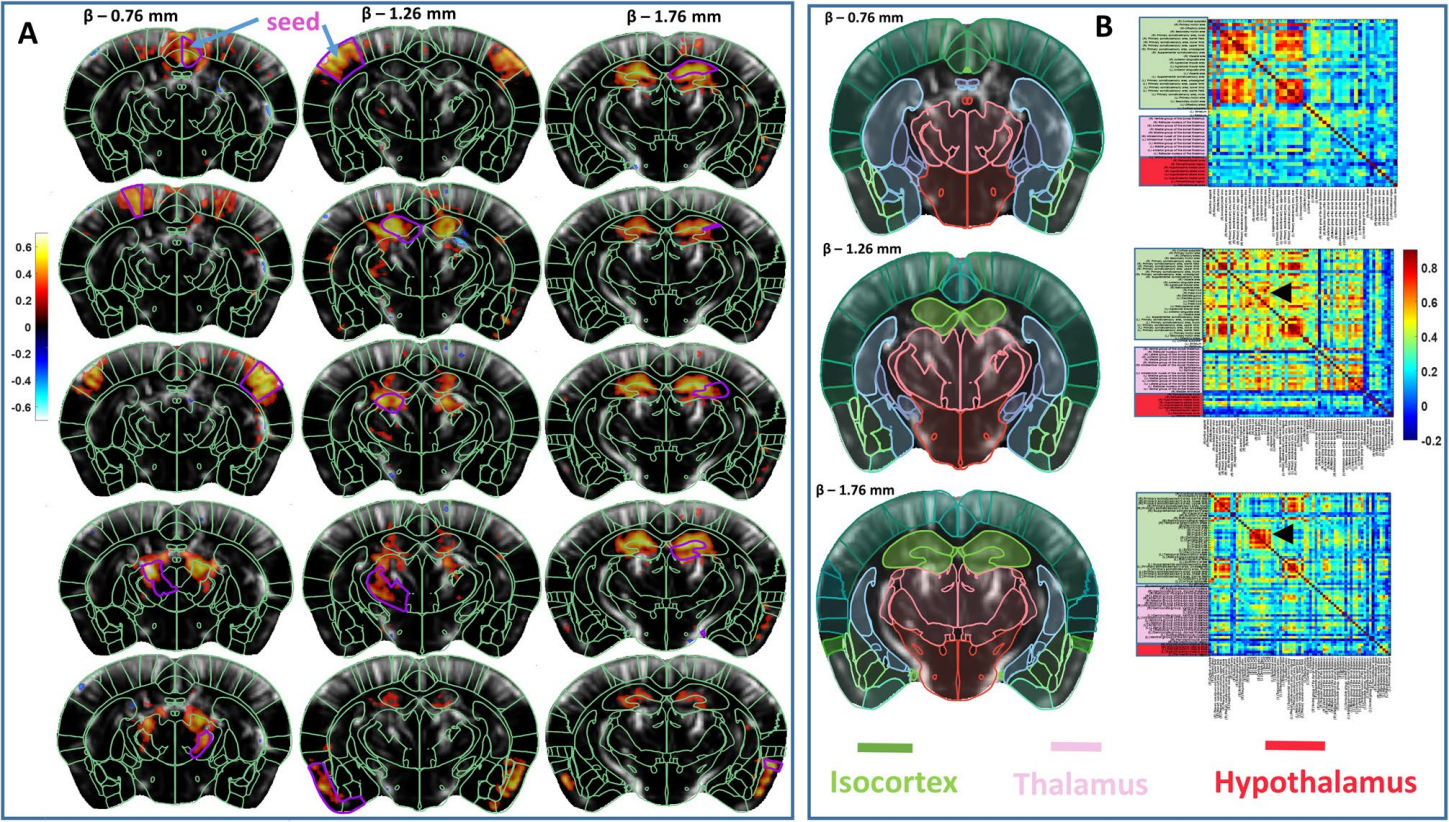
Transcranial functional connectivity analysis with BPS and atlas-based segmentation. (**A**) Seed-based analysis. The grayscale images represent the baseline Power Doppler images. Seed regions are indicated in magenta. Color-coded correlation maps are overlaid to baseline images for each of the selected seed regions. Automatically aligned anatomic delineations from the Allen CCF are shown in green for reference. (**B**) Connectivity matrix analysis. BPS enabled anatomic boundaries delineation and data extraction from about 70 regions for each of the 3 imaging planes. Regions are overlaid onto the baseline Power Doppler images. Connectivity matrices were computed based on pairwise correlations between signals from individual regions. The matrices show color-coded correlation coefficients. Arrowheads in (**B**) indicate connections within the hippocampal formation.

Figure 7A shows a seed-map overlaid onto the baseline Power Doppler images and the functional regions (from Allen Atlas) automatically aligned. The seed region is indicated with a different color. At Bregma − 1.76 mm position we observed high correlation within hippocampal formation and a good overlap between the automatically aligned regions and the seed map. Cortical bilateral connections are also observed at Bregma − 1.26 mm and Bregma − 0.76 mm, as well as deeper structures such as thalamus or olfactory areas.

The connectivity matrix over 50 regions (for a single slice) extracted from the Allen Atlas allows observation of robust connectivity between interhemispheric functional regions as well as higher connectivity within main functional areas (Fig. 7B), as previously observed in fUS connectivity analysis with manually positioned regions in the literature.

## Discussion

Functional ultrasound with ultrafast Doppler imaging is capable of mapping and recording blood flow variations and allows functional activation mapping and connectivity analysis. Here we demonstrate that 3D Power Doppler vascular fingerprints can be automatically registered ‘on the fly’ to provide automatic neuronavigation for live probe positioning and atlas-based data analysis. Using angiographic images obtained with Ultrasound Localization Microscopy, we demonstrated the vascular registration accuracy to be less than 100 µm, i.e. the size of the Power Doppler pixel, and more accurate than the manual landmark annotations of experts.

The BPS appears to rely mostly on larger vessels distributed throughout the brain rather than small vessels, which is consistent with the fact that smaller vessels may be more subject to change between individual mice. We believe that local variations between vessel architecture cancel out overall in the whole brain, which allows the registration to remain accurate in similar strains of mice. These invariants of vascular shapes versus scale relationships will be further studied in subsequent work.

We demonstrated the use of the BPS for functional ultrasound imaging in complex oblique planes automatically, with a good match between activated areas and atlas-based structures, as well as functional connectivity matrix assessment with automatic extraction of ROIs for any plane.

Our study has several limitations. First of all, all mice used in this study are from the strain C57BL/6 and aged between 7 and 14 weeks. The ability to use the same vascular reference for mice with different ages, for different models, or for different pathologies, has to be further investigated and it might be necessary to use a different ad-hoc vascular template adapted to each category. On the processing side, the registration process was also limited to a simple affine registration algorithm^27^. However, it would be straightforward to include non-rigid deformable registration based on (for instance) deformable demons^30,31^, B-spline^32^ or thin-plate spline^33^ registration, which might be required in more complex applications such as neurodevelopmental studies, or to take into account large brain deformation for instance due to a craniotomy (brain shift), large cerebral tumor growth or cerebral oedema/brain swelling. This approach could also be required for animal models with less genetic homogeneity, which could yield more diverse brain and vasculature shapes across subjects. This would certainly be the case for functional ultrasound imaging with BPS in primates^34^ or humans, in the case of intraoperative^35,36^ settings or neonate imaging^37,38^, where there can be a high natural variability between subject brains.

In this work, the 3D Doppler reference was pre-aligned to the Allen template to illustrate the BPS approach on mice, but other atlases or templates could be used, for instance for other animal species. The alignment between the Allen template and the Doppler was performed through an existing MRI–microCT angiography dataset, but alignment using MRI angiography is also being explored, with good preliminary results. Using MRI angiography would allow more specific alignment especially for specific strains, age group or other animal species.

Although recent promising advances in the development of 2D matrix arrays or row column arrays have been proposed for full 3D functional ultrasound imaging^6–8^, their sensitivity remains limited, which still precludes transcranial imaging in mice. The proposed approach still remains valid for volumetric imaging even if the need to perfectly position the probe prior to the acquisition is reduced, as there remain more possibilities to register volumes in post-processing.

Another application of the on-the-fly BPS approach is the use of the Doppler images and a robotic motorized platform to allow guidance of tools deep in the brain such as needles for micro-injections, micro- or array electrodes for recording, or optical fibers for optogenetic stimulation in deep areas. This could also be a promising tool for human surgery and the positioning of electrodes for deep brain stimulation, especially in conjunction with live functional ultrasound imaging.

Finally, the super-resolution vascular images were used here only as a means to investigate the registration accuracy, but they could one day be used as a second step to further refine the registration to the tens of micrometer scale, enabling extremely precise neuronavigation in depth in a unique way.

The brain vascular GPS approach presented here enables on-the-fly complex brain navigation without neuroanatomic expertise, in the context of functional ultrasound acquisition and data analysis with more accurate positioning and identification of regions of interest. It could help with the standardization of acquisitions in standardized planes to allow the building of large, reproducible and reliable fUS datasets and studies. Beyond mice studies, the BPS proposed here can be generalized to many other animal models including rats, ferrets, marmosets and non-human primates, as well as to clinical applications such as transcranial imaging^22^, neurosurgery guidance or neonate functional imaging.

## Supplementary Information

Supplementary Figures.

## Data Availability

Data supporting the findings of this study are available in the framework of an official collaboration between academic institutions.
