# Supplementary material for: A functional ultrasound brain GPS for automatic vascular-based neuronavigation
Source: Sci Rep. 2021 Jul 26;11:15197. doi: 10.1038/s41598-021-94764-7 (PMC8313708; doi:10.1038/s41598-021-94764-7)
Supplement: Supplementary file 1 — Supplementary Figures. [file 41598_2021_94764_MOESM1_ESM.pdf]

A functional ultrasound brain GPS for automatic vascular-based neuronavigation

M. Nouhoum[1,2], J. Ferrier[2], B.-F. Osmanski[2], N. Ialy-Radio, S. Pezet[1], M. Tanter[1], T. Deffieux[1*]

1. Physics for Medicine, INSERM U1273, ESPCI Paris, CNRS UMR 8063, PSL Research University, 17 rue Moreau, Paris, France.

2. Iconeus, 6 rue Jean Calvin, Paris, France.

* Corresponding author.

Supplementary 1

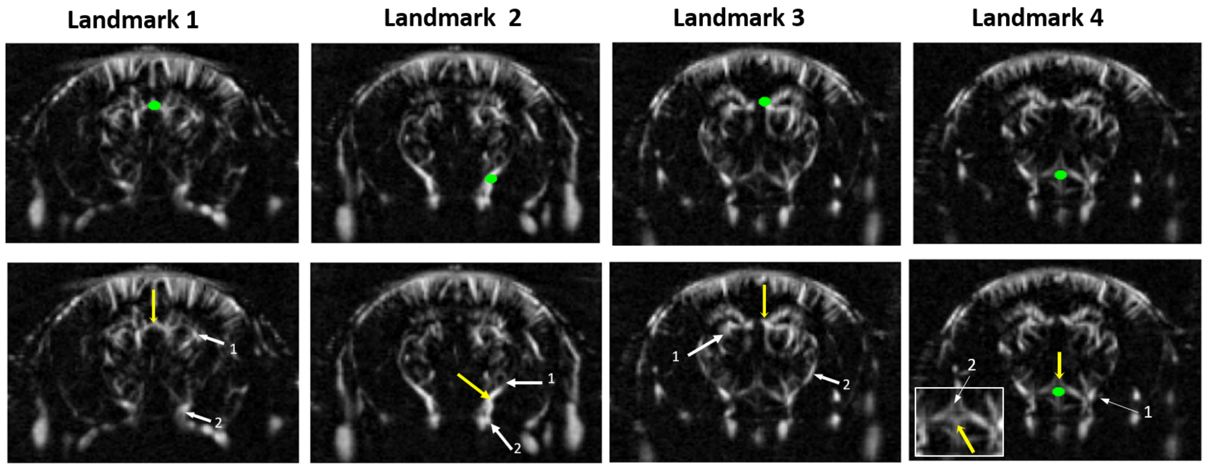

Supplementary 2

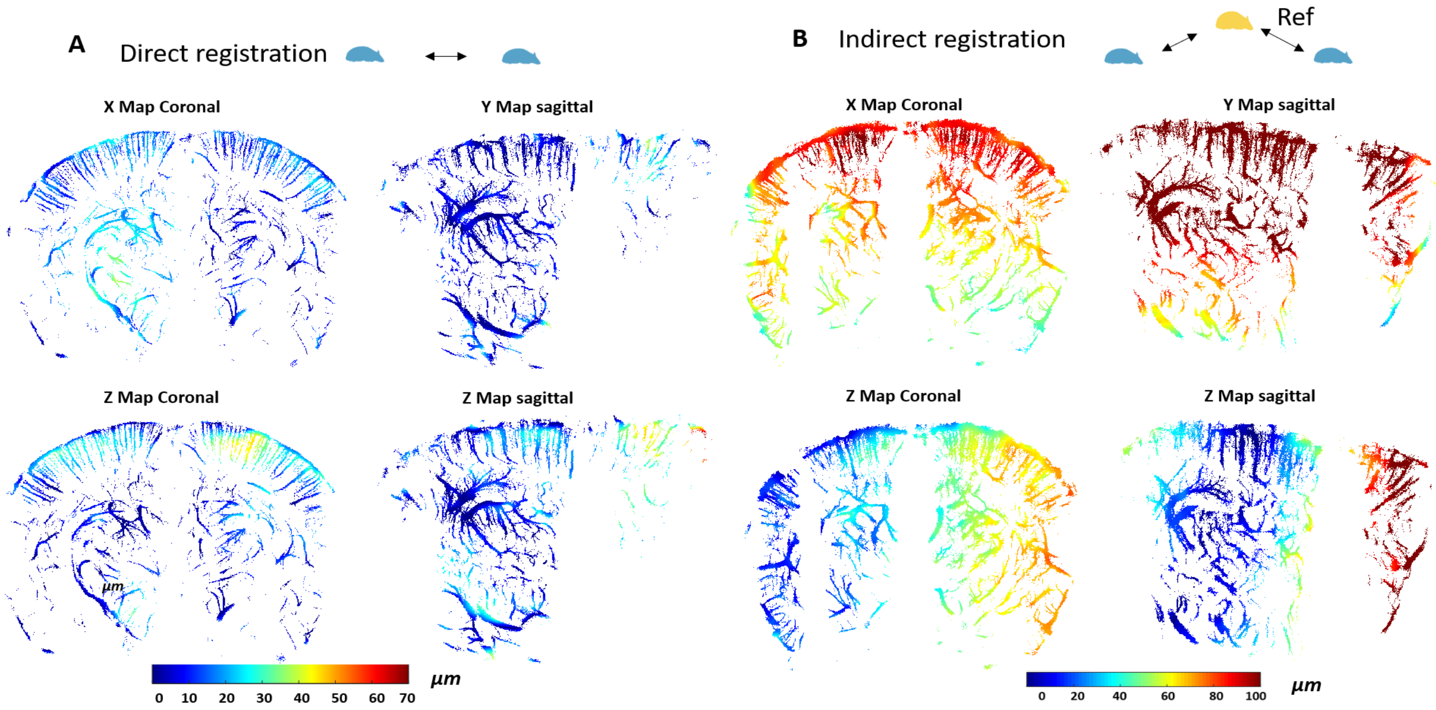

Supplementary figure 1 : Definition of the vascular landmarks defined by the neurobiologists. Definition of the 4 landmarks (yellow arrow) and vascular landmarks used to recognize the imaging plane.

Supplementary figure 2 : Displacement map from non-rigid registration of pair of super resolution images. A. Shifts of the coronal and sagittal images obtained after positioning guided by direct registration. We obtain lateral (X) and axial (Z) displacement fields with the coronal slices and transverse (Y) and axial (Z) displacement fields with the sagittal slices. **B.** Same as A. after positioning guided by direct registration
